# Immobilization of **α**-Amylase onto *Luffa operculata* Fibers

**DOI:** 10.1155/2013/803415

**Published:** 2013-03-31

**Authors:** Ricardo R. Morais, Aline M. Pascoal, Samantha S. Caramori, Flavio M. Lopes, Kátia F. Fernandes

**Affiliations:** ^1^Laboratório de Química de Proteínas, Instituto de Ciências Biológicas, Universidade Federal de Goiás, Cx. Postal 131, 74001-970 Goiânia, GO, Brazil; ^2^Unidade Universitária de Ciências Exatas e Tecnológicas, Universidade Estadual de Goiás, Rodovia BR 153, Km 98, Cx. Postal 459, 75132-903 Anápolis, GO, Brazil; ^3^Faculdade de Farmácia, Universidade Federal de Goiás, Avenida Universitária com 1a Avenida, Setor Universitário, 74605-220 Goiânia, GO, Brazil

## Abstract

A commercial amylase (amy) was immobilized by adsorption onto *Luffa operculata* fibers (LOFs). The derivative LOF-amy presented capacity to hydrolyze starch continuously and repeatedly for over three weeks, preserving more than 80% of the initial activity. This system hydrolyzed more than 97% of starch during 5 min, at room temperature. LOF-amy was capable to hydrolyze starch from different sources, such as maize (93.96%), wheat (85.24%), and cassava (79.03%). A semi-industrial scale reactor containing LOF-amy was prepared and showed the same yield of the laboratory-scale system. After five cycles of reuse, the LOF-amy reactor preserved over 80% of the initial amylase activity. Additionally, the LOF-amy was capable to operate as a kitchen grease trap component in a real situation during 30 days, preserving 30% of their initial amylase activity.

## 1. Introduction

Amylases figure among the most studied enzymes for biotechnology and industrial purposes [1, 2]. They are used in many industrial fields, including food, detergent, textile, and paper industries [3–5]. 

A main problem of food industries, particularly bakeries, pastries, and industrial kitchens, is the starch waste produced from machines, cans, and containers washing. This waste can obstruct industrial ducts causing several damages and financial losses.

An alternative to avoid these losses is the continuous use of detergents containing high concentration of amylase. Nevertheless, they are more expensive than the nonenzymatic detergents, making them not attractive and impracticable for small business.

To solve these inconvenient, immobilized enzymes may play a remarkable role, once their main advantage is the repeated use, which considerably reduces the costs [6–8]. 

Among the several available methods of immobilization, adsorption is the cheapest one considering the reagents and time employed [9–13]. This method should be considered when the costs of the final product are a limiting factor for the process. 

For this purpose, the material of the support must be considered, once its destination cannot cause environmental damages. Natural organic materials are promising candidates for large-scale processes, because of their biodegradability behavior, permitting the use of these substances as clean devices in the environment [14]. 

In this study, a commercial thermostable *α*-amylase was immobilized by adsorption onto *Luffa operculata* fibers (LOFs). The system LOF-amy was used for continuous starch hydrolysis and wastewater treatment in kitchen grease traps.

## 2. Material and Methods 

*Luffa operculata* L. fruits were collected in the city of Goiânia, GO (Brazil). The enzyme used for immobilization was an amylase (6400 U mL^−1^) produced by *Bacillus licheniformis* (Resamylase, Tecpon, RS, Brazil). All chemicals obtained were from analytical purity.

### 2.1. Support Preparation

*Luffa operculata* L. fruits were manually decorticated, the seeds were removed, and the remaining fibers were soaked in tap water for 12 h, or until the water became clear. The washed fibers were dried and stored in polypropylene bags, at room temperature, until its use.

### 2.2. Enzyme Activity

Before immobilization, the enzyme solution was filtered (Whatman n. 1) and diluted in distilled water at proportion of 1 : 50. The enzyme activity was measured according to methodology described by Fuwa [15], based on the iodine-starch colored complex, as follows. To 20 *μ*L of free amylase or 125 mg of LOF-amy were added 80 *μ*L of 0.1 mol L^−1^ phosphate buffer pH 7.5. Following, 100 *μ*L of 0.5% (w/v) potato starch were added, and the mixture was incubated at 37°C, during 15 min. The reaction was stopped by the addition of 200 *μ*L of 0.1 mol L^−1^ acetic acid and 200 *μ*L of Fuwa reagent. The volume of each replicate was adjusted to 10 mL using distilled water, homogenized, and measured at 660 nm (Ultrospec 2000, Pharmacia). Amylase activity was expressed in terms of starch hydrolysis. One unit of enzyme was the amount of enzyme that hydrolyzes 1 *μ*g starch in the reaction time. The values obtained in blank reactions performed in the presence of starch but without amylase, or in the presence of starch and LOF without adsorbed amylase, were discounted from all readings.

### 2.3. Enzyme Immobilization

In order to determine the retention capacity of *Luffa operculata* fibers (LOFs) to retain amylase, immobilization tests were conducted varying the amount of offered enzyme (38.5 U, 77 U, and 154 U). In a typical test, 1.2 mL of amylase solution was left to react with 125 mg of LOF, for 12 h, at 4°C. Following, the LOF was removed from amylase solution, submerged for 1 min in a 0.1 mol L^−1^ phosphate buffer, pH 7.5 (four times), and then dried at room temperature. The pieces of LOF containing adsorbed amylase (LOF-amy) were stored at room temperature, in polypropylene flasks, until its use for starch hydrolysis.

Total immobilized protein was measured in order to calculate the specific retained activity. The difference between the soluble protein in the native enzyme solution and the supernatant after immobilization was used to estimate the content of immobilized protein. Activities of the native and immobilized amylase (LOF-amy) were measured according to methodology. 

### 2.4. Characterization of LOF-Amy

The optimum assay temperature was determined incubating LOF-amy with starch solution at 25, 28, 30, and 40°C, for 15 min. After that, the LOF-amy was removed from the bulk, and the supernatant received acetic acid and Fuwa reagent. This solution was diluted as described in item 3.3 and read at 660 nm.

In order to determine the time necessary for complete hydrolysis of 0.5% (w/v) starch solution, reactions were performed during 5, 10, and 15 min, at 37°C. After removing LOF-amy fragments, the supernatant received acetic acid and Fuwa reagent. The hydrolytic capacity of LOF-amy was tested using 0.5%, 1%, 1.5%, and 2% (w/v) starch solutions. 

### 2.5. Stability of LOF-Amy

The shelf life of LOF-amy was determined by the storage of the dried support containing immobilized enzyme, at 4°C. Each 7 days of storage, a new sample of LOF-amy was tested for amylase activity. 

Operational stability, defined as the combination of storage and repeated use, was tested using the LOF-amy samples stored at 4°C. Each 7 days of storage, a fragment of LOF-amy was used for starch hydrolysis. After assay, the sample of LOF-amy was washed with 0.1 mol L^−1^ phosphate buffer, pH 7.5, to remove reaction residues, dried at room temperature, and again stored at 4°C until new hydrolysis test. 

### 2.6. Using Different Substrates

Aiming to verify the ability of LOF-amy in hydrolyze starches from different sources, tests were conducted using 0.5% (w/v) solutions of cassava starch, maize starch, and wheat starch. Blanks were made for each starch and discounted from readings. The hydrolysis capacity was determined as item 3.3.

### 2.7. The LOF-Amy Reactor

**Figure 1:**
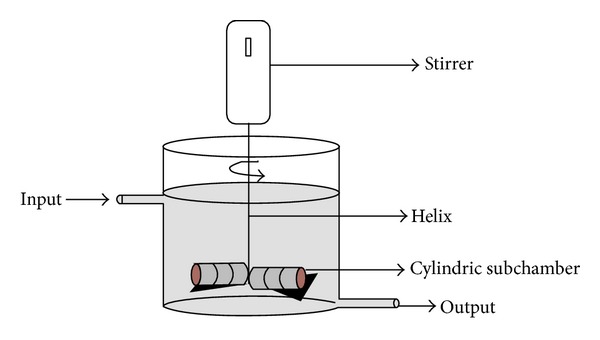
The LOF-amy reactor. The cylindric subchambers containing LOF-amy samples are shown.

The batch reactor was built in a polypropylene reaction chamber with a maximum capacity of 2 L containing a central helix, which promoted the homogenization through a radial rotation (Figure 1). The helix supported perforated cylindrical subchambers containing LOF-amy, ranging from 1.0 to 7.0 g. During a hydrolytic reaction, the moving of the helix allowed the substrate diffusion to the LOF-amy. A typical reaction was performed adding 1 L of 0.5% starch solution (w/v) and the hydrolysis of starch proceeded as item 3.3. Aliquots were collected each 15 min of reaction and starch hydrolysis measured as described. Results were presented as percentage of remaining activity compared to the first use.

### 2.8. Use of LOF-Amy in Grease Traps

The LOF-amy fragments were used as kitchen grease trap component. During 30 days the system was used continuously in a real situation. After that, the fragments were removed from grease trap, cleaned with distilled water, and the residual amylase activity stability measured as described. 

### 2.9. Statistical Analysis

All experiments were conducted in triplicates and mean values were reported. Statistica software (Statistica 6.0, StatSoft Inc., OK, USA) was used to perform analysis of variance (ANOVA) followed by the Tukey test to determine the significant differences among the means. The level of significance used was 95%.

## 3. Results and Discussion

**Figure 2:**
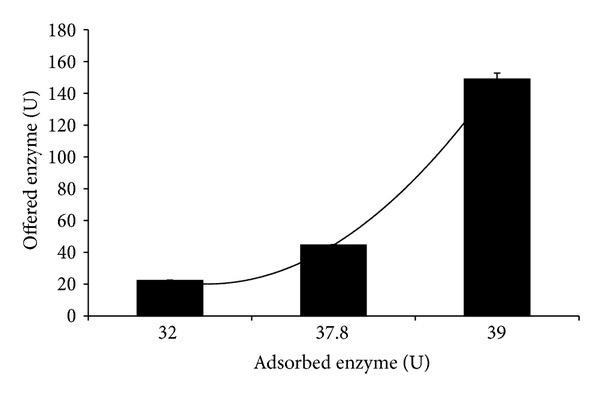
Relationship between amounts of amylase offered and amounts immobilized (*r* = 0.9762).

The relationship between the amount of enzyme offered and amount of enzyme immobilized is in close agreement with an adsorption process. As can be seen in Figure 2, the system presented exponential tendency (*r* = 0.99). The relation between the concentration of the enzyme in the solution and the amount adsorbed to LOF describes a type III adsorption isotherm, characteristic of a multiple layers system, which will drive the equilibrium. In this sense, increasing the amount of enzyme offered will displace the adsorption equilibrium in direction of the support adsorption. 

**Figure 3:**
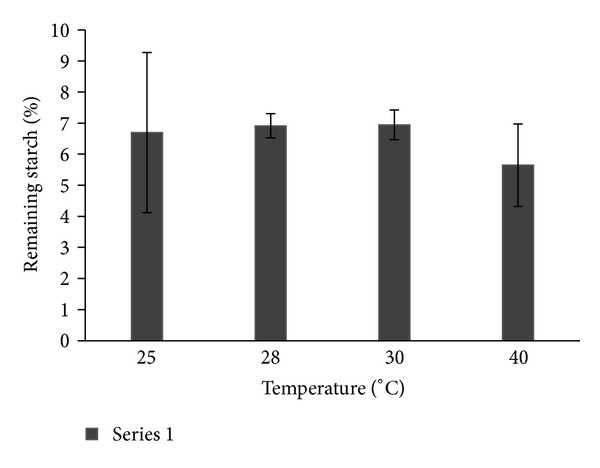
Optimum assay temperature for LOF-amy.

Some reaction parameters were tested to verify if immobilization affected amylase activity. Concerning assay temperature (Figure 3), there was no significant difference in the amylase activity in the tested interval (25–40°C). The thermal stability, the more important characteristic of this commercial amylase, was preserved in the immobilization process. This temperature range has been chosen due to practical application since this is the range of the waste water that frequently reaches the grease traps. In this sense, the maintenance of thermal stability was a very good result. 

**Figure 4:**
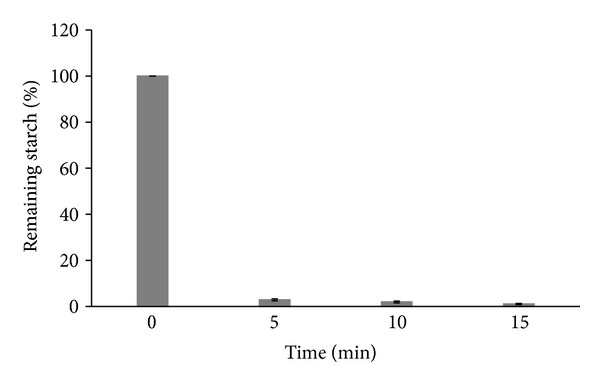
Time course for starch hydrolysis by LOF-amy.

The time course of the reaction performed by LOF-amy is presented in Figure 4. Increasing the reaction time resulted in increases in the hydrolysis rate, with 97.4%, 98%, and 98.9% hydrolysis after 5, 10, and 15 min, respectively. After optimization of the immobilization parameters, 32 U were immobilized onto 125 mg of LOF, corresponding to 0.26 U mg^−1^ and an efficiency of 83.1%.

**Figure 5:**
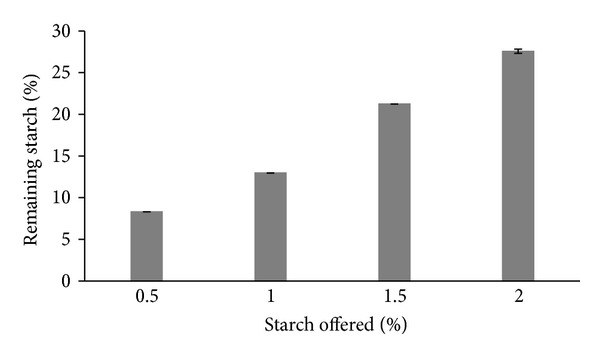
Maximum starch hydrolysis produced by LOF-amy (*r* = 0.9861).

The hydrolytic capacity of LOF-amy is shown in Figure 5. The kinetics of starch hydrolysis as function of increasing amounts of substrate from 0.5 to 2.0% (w/v) was linear (*r* = 0.986). It means that LOF-amy is operating in its initial velocity, and the system is out of the saturation zone. Enzymatic industrial reactors for starch hydrolysis are designed to operate with starch concentrations in the range from 2 to 10% (w/v) and temperatures around 50°C [16]. The system LOF-amy seems to be able to operate in conditions very near of these industrial conditions.

The shelf life of LOF-amy was tested during three weeks (Table 1). As can be seen, after one week stored at 4°C there was 20% of activity loss. After that, no further loss in activity was detected. The activity loss observed probably occurred during drying procedure, when the evaporation of water molecules induced rearrangements of the polypeptide chain. These rearrangements may occur in different patterns for amylase molecules as function of the specific surrounding environment. In this case, when rehydrated, some molecules did not recover their original tridimensional structure, and hence, did not recover their hydrolytic activity. Cordeiro et al. [1] stored dried *Bacillus licheniformis* amylase immobilized onto polyethylene alt-maleic anhydride for 24 and 48 h, at room temperature. After rehydration with phosphate buffer, the authors observed 31% to 48% of activity loss for samples stored for 24 h and 53%–62% of activity loss after 48 h of storage.

**Figure 6:**
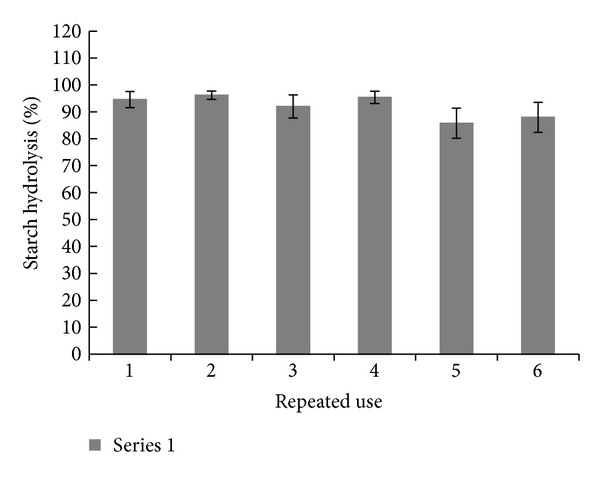
Operational stability of LOF-amy.

The operational stability LOF-amy is presented in Figure 6. After six cycles of use and washing the system LOF-amy retained 92% of initial activity. This result was similar to some successful immobilized systems, such as amylase immobilized in copolymers of methacrylate-acrylate acid that preserved 95% of initial activity after five cycles [17]. On the other hand, entrapment of amylase in calcium alginate beads retained 60% of initial efficiency after five batches of use [16], and covalent attachment of amylase onto chitosan and amberlite resulted in losses of 40% and 30% after the fourth use, respectively [18]. It is outstanding that in these systems, the methodology used for enzyme immobilization was covalent bound or entrapments, which are methodologies where the forces involved in enzyme-support bounding are higher than those present in adsorption.

The ability to hydrolyze starches from natural sources was tested for LOF-amy. This assay is very important considering the possible practical applications. Many authors have shown high amylase performance only under controlled conditions and single substrate source [19–21]. Nevertheless, if practical applications are considered, it is very important to analyze the enzyme behavior against their natural substrates. After immobilization on *Luffa operculata* fibers, amylase preserved its hydrolytic capacity over different substrates. Maize starch was better hydrolyzed by this system (93.96% ± 0.55), followed by wheat starch (85.24% ± 3.97) and cassava starch (79.03% ± 6.24).

**Figure 7:**
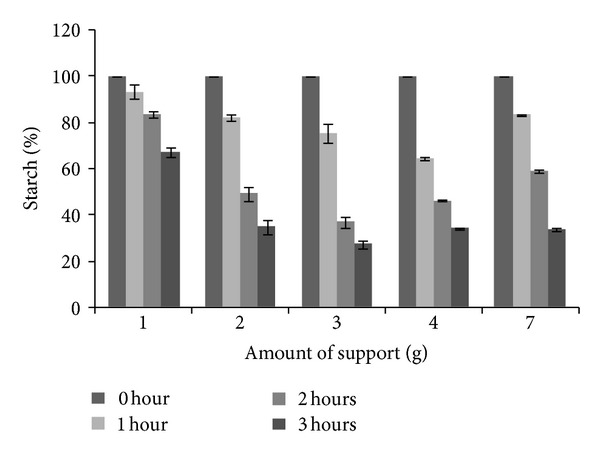
Starch hydrolysis capacity by LOF-amy reactor. Data were expressed as mean ± standard deviation. The starch hydrolysis was monitored during 3 h.

Attempting to optimize starch hydrolysis in a 2 L reactor, reactions were conducted varying the amount of LOF-amy inside the kitchen grease trap. Results are shown in Figure 7.

The best performance was observed when subchamber was filled with 3.0 g of LOF-amy and a reaction time course of 3 h. In this condition, 71.9% of starch hydrolysis was reached. The reactors with 2.0, 4.0, and 7.0 g of the LOF-amy did not present significant differences among them, showing approximately 64.9% of hydrolysis yield after 3 h reaction.

**Figure 8:**
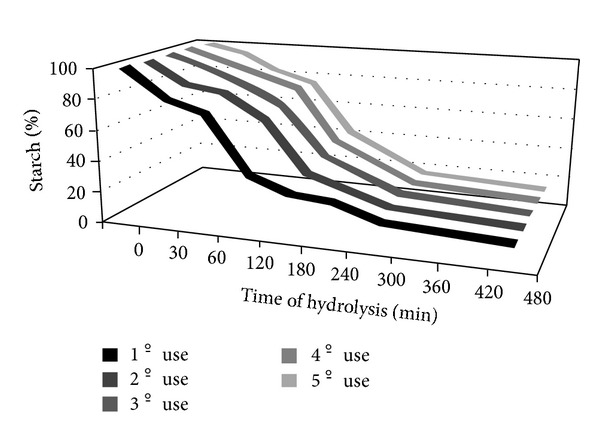
Operational stability of LOF-amy reactor.

Under reactor operational conditions, LOF-amy was able to hydrolyze starch during a long period (300 min) for five batch operations. Figure 8 displays a typical course of an endoamylase reaction. Initially the rate of starch disappearing is apparently low because the fragments generated in hydrolytic reaction are still able to react with iodine from Fuwa reactive, producing colored product. After 120 min, the hydrolysis rate presents an apparent increase. This may be explained because in this step, the starch fragments (oligosaccharides) became small enough not to react with iodine. In this point, it was possible to observe 62.5% of starch hydrolysis. After this point, the hydrolysis rate starts to show the effect of substrate limiting in the reaction medium. At the end, all the starch breakdown kinetic curves presented similar behavior (82.46% at 300 min).

The high chemical and biochemical oxygen demands in the wastewater nontreated samples require the development of clean technologies for effluents treatment [22]. The kitchen grease traps containing LOF-amy showed 30% of remaining activity after 30 days of continuous use. Cammarota and Freire [23], in a review about the role of hydrolytic enzymes in the treatment of wastewater, discussed the disadvantages of pretreatment methods, such as grease traps. The high cost of the reagents used in these systems was considered as a problem to be solved. The LOF-amy developed here presents, at least, two advantages. First, *α*-amylase immobilization allowed the enzyme reusability, even under nonoptimal conditions. Second, the same immobilized system can be used over a long period. Those characteristics associated to the ease of system production and the use of biodegradable materials may be a potential tool to be chosen for water treatment purposes.

## 4. Conclusions

A commercial amylase was successfully immobilized by adsorption of a natural support. The system presented great performance and stability, compared to the covalent attach immobilization current derivatives. LOF-amy system could operate under continuous starch hydrolysis, and their use for waste starch degradation was obtained for over 30 days. This nonexpensive, ease handing material can be a good choice of immobilized amylase large-scale application.

## Figures and Tables

**Table 1:** LOF-amy remaining activity after storage for three weeks.

Storage time (weeks)	Residual activity (%)
0	100
1	80.67 ± 18.64
2	79.36 ± 23.27
3	86.42 ± 13.97

Data was expressed as mean ± standard deviation.
